# Fueling the Covid-19 pandemic: summer school holidays and incidence rates in German districts

**DOI:** 10.1093/pubmed/fdab080

**Published:** 2021-03-26

**Authors:** Thomas Plümper, Eric Neumayer

**Affiliations:** Department of Socioeconomics, Vienna University of Economics and Business, Vienna 1020, Austria; Department of Geography and Environment, London School of Economics and Political Science (LSE), London WC2A 2AE, UK

**Keywords:** infectious disease, public health, communicable disease

## Abstract

**Background:**

The Robert-Koch-Institute reports that during the summer holiday period a foreign country is stated as the most likely place of infection for an average of 27 and a maximum of 49% of new SARS-CoV-2 infections in Germany.

**Methods:**

Cross-sectional study on observational data. In Germany, summer school holidays are coordinated between states and spread out over 13 weeks. Employing a dynamic model with district fixed effects, we analyze the association between these holidays and weekly incidence rates across 401 German districts.

**Results:**

We find effects of the holiday period of around 45% of the average district incidence rates in Germany during their respective final week of holidays and the 2 weeks after holidays end. Western states tend to experience stronger effects than Eastern states. We also find statistically significant interaction effects of school holidays with per capita taxable income and the share of foreign residents in a district’s population.

**Conclusions:**

Our results suggest that changed behavior during the holiday season accelerated the pandemic and made it considerably more difficult for public health authorities to contain the spread of the virus by means of contact tracing. Germany’s public health authorities did not prepare adequately for this acceleration.

## Introduction

Holiday travels can be expected to accelerate the SARS-CoV-2 pandemic. To a small extent, this is because traveling via bus, train or plane adds to the risk of becoming infected.^1^^,^^2^ More importantly, infections rise because individuals change their social behavior during holidays.^3^ Many holiday-makers have more and more intense social interactions, often to people that they do not know and do not share social capital with which has been found to be conducive to maintaining social distancing norms.^4^^,^^5^ Mobility also reduces the health agencies’ ability to successfully trace close contacts of people that are infected with Sars-CoV-2.

The Robert-Koch-Institute (RKI) reports that over Germany’s entire summer school holiday period in ~27% of weekly cases reported to the Institute a foreign country was mentioned as the most likely place of infection.^6^ This figure reached its maximum at 49% of weekly cases in week 34, which is in mid-August. It is, however, not possible to interpret these numbers as the effect of holiday-related travel since some of the infections may not actually have occurred abroad despite ‘abroad’ being mentioned as the likely place of infection, not all international travel is necessarily holiday-related even if it takes place during the holiday season and not all holiday-makers spend their holidays abroad. The RKI numbers shed light on the relevance of international travels for the epidemic situation in Germany, but they may over-state or under-state the true impact of the holiday season on incidence rates.

We complement the RKI’s analysis by studying the extent to which summer school holidays have accelerated the pandemic in Germany. In order to estimate the effect of summer school holidays on the weekly incidence rate, we employ an ecological analysis of variation in the weekly SARS-CoV-2 confirmed case incidence rate across German districts (individual-level data do not exist). Germany provides an excellent case study since we can exploit a particular feature of its system of school holidays, namely that they are not uniform across the Federal Republic but vary in their start and therefore also their end date from state to state in a pre-determined way. This idiosyncratic feature allows us to disentangle the effect that holidays have had on incidence rates in German districts located in states that are or have been on holiday from the general upward trend in new infections in Germany.

We test the following four hypotheses: First, school holidays have a positive effect on incidence rates. Second, the later parts of any given holiday season have a larger effect than its earlier parts given that there is a delay until holiday travelers return home and given infections are on the rise in practically all holiday travel destinations, both within and outside Germany, thus increasing the risk of catching the virus as the holiday season proceeds. Third, the holiday season does not merely increase individual risks. Travel associated with the holiday season should also have a lasting effect on the epidemic situation in the home districts because any infected returning traveler increases the probability of additional infections. Thus, the effects of holidays and holiday-related travel on incidence rates do not disappear when the holiday season is over. And fourth, school holidays will have a stronger effect on incidence rates in districts that are richer on average and in which a larger share of the resident population are foreigners. Richer people can afford better to go on holiday for longer and foreign citizens are likely to use the holiday season for returning to their home country for family visits (possibly in addition to taking other holidays) not least because the lockdown in the spring of 2020 prevented most of them from seeing family abroad over the Easter holiday period.

There is surprisingly little existing evidence on the impact of public holidays on the SARS-CoV-2 pandemic. Early research has shown that the extension of the Lunar New Year holidays in China has contributed to the country’s successful containment of the pandemic.^7^ However, it is probably impossible to generalize these findings because the pandemic started around the time of this holiday period and the holiday extension helped authorities to identify infected individuals before traveling home. Two other studies point in the opposite direction, suggesting that Israel’s hitherto successful mitigation policy broke down in the wake of mass social gatherings during the 9–11 March Jewish holiday of Purim or that holiday-related travels from metropolitan areas to the provinces in Sweden may spread infections.^8^^,^^9^ To the best of our knowledge, our is the first academic study of the impact that the summer school holiday season has actually had on the pandemic.

## Material and Methods

### Material

Our dependent variable is the weekly incidence rate (per 100 000 people) in a German district. Data are sourced from the RKI website (www.rki.de). They are based on confirmed positive tested cases. While the number of confirmed cases can be a problematic measure for the pandemic’s dynamics, we know of no reason why testing would systematically vary across German districts.

Our sample covers all 401 districts in Germany with the 12 districts of Berlin aggregated to one single city state district due to lack of disaggregated data on the conditioning variables employed for testing one of our hypotheses. The temporal dimension is drawn from the period starting with the weekly incidence rate on Wednesday 10 June (week 23) and terminating with the weekly incidence rate on Wednesday 23 September (week 38). We deliberately define the week to end in a Wednesday rather than Sunday or Monday to avoid noise from occasional corrections made on Mondays or Tuesdays to compensate for under-reporting to the RKI over the weekend. For each district, we analyze the period ranging from 2 weeks prior to the beginning of holidays to 2 weeks after the end of the holidays. Our panel thus has *N* = 401 districts and *T* = 10 weeks equals 4010 observations.

In Germany, the dates of the summer school holidays are chosen years in advance by each of the 16 states in close consultation with each other. The intention is to reduce the probability and length of traffic jams on Germany’s crowded motorways during the summer months. In each state, schools close for ~6 weeks. In 2020, the summer school holiday season began on June 22 in Mecklenburg-Vorpommern and ended September 9 in Baden-Württemberg. Hence, Germany spreads the holidays over almost 13 weeks (see the Supplementary Document for full overview).

### Methods

The average weekly incidence rate across all German districts over the entire sample period is 6.38 cases per 100 000 people with a standard deviation of 10.14. In Germany as a whole, the number of new infections had been stable at around 500 per day until the end of July. In August, the number of daily confirmed cases begun to rise reaching ~1500 new cases at the end of August and ~2000 new daily cases at the end of September.

This upward trajectory in Germany coincides with the summer school holiday season. It is unlikely, however, that the return of rising incidence rates has been determined by school holidays alone. To isolate the predicted effects from other influences, we include a lagged dependent variable to account for the common trend in the data.^10^ Results are similar if we use an alternative approach for taking out the common trend, such as an autoregressive model of order one (results not reported). This is a conservative research strategy since part of this trend was most likely caused by returning holiday-makers. However, it is impossible to provide a precise estimate of the influence of holidays on the common trend because holiday travel was allowed in all states and all districts at all times, not just during school holidays.

Most of our estimation models are based on a specification with a dummy variable that is set to 1 if a district is located in a state in which schools are on summer holidays in that week as well as a dummy variable for the 2-week period after the holidays. This specification can be interpreted as a Chow-type model,^11^^,^^12^ in which the dummy variables estimate whether there is a structural break between the holiday period as well as the period of 2 weeks after the holidays end, both relative to the period of 2 weeks before holidays begin, the presumed counterfactual.

This relatively simple specification with two dummy variables only is handy for extensions where we allow their effect to vary by state and allow their effect to be conditioned by two district-level variables that are likely to impact on the number of holiday-related travels undertaken from each district (on which more below). It is not an optimal specification however given it presumes the effect to be constant within the holiday period. Empirical evidence suggests that the average length of holiday stay of German tourists is ~12–14 days.^13^ Therefore, infections should start to rise only ~2–4 weeks after the beginning of the school holidays. Therefore, we will also present results from a more appropriately specified model that allows the effect of the holiday period to vary week-by-week.

We estimate our models with a linear fixed effects estimator that absorbs any variation across districts that is time-invariant such as demographic, geographic and socio-economic factors that render some districts more generally exposed to the pandemic than others.^14^ If we estimate the models with Arellano and Bond’s dynamic panel estimator instead, results are very similar (results not reported).^15^ Standard errors are clustered on districts. If we additionally apply two-way clustering of standard errors also by states results are hardly affected (results not reported). Since potential control variables come from annual data, they are time-invariant for the specific panel structure we have. These time-invariant variables are perfectly collinear with the district fixed effects and we therefore cannot estimate their effect in a district fixed effects model. They can however condition the effect of the time-varying school holidays variables, as we do in one model employing average taxable income and the share of foreigners amongst a district’s resident population as conditioning variables.

## Results

In Table 1, we first of all report results on a dummy variable that is set to 1 if a district is located in a state in which schools are on summer holidays in that week as well as a dummy variable for the 2-week period after the holidays (model 1). We find that the summer school holiday weeks are on average predicted to increase incidence rates by 1.71 cases per 100 000 people relative to the period before holidays, consistent with our first hypothesis. The 2-week period after holidays end is predicted to increase incidence rates by 4.81 cases per 100 000 people, consistent with our third hypothesis.

**Table 1:** School holiday effects, pooled and time-varying

	*Model 1*	*Model 2*
Incidence rate (*t* − 1)	0.390^***^	0.366^***^
	(12.17)	(13.68)
Summer school holidays dummy	1.712^***^	
	(4.621)	
2 weeks after summer school holidays dummy	4.811^***^	
	(14.04)	
		
First week of summer school holidays		0.253
		(0.429)
Second week of summer school holidays		−0.223
		(−0.557)
Third week of summer school holidays		0.814
		(1.471)
Fourth week of summer school holidays		2.666^***^
		(5.559)
Fifth week of summer school holidays		3.003^***^
		(6.626)
Sixth week of summer school holidays		4.145^***^
		(9.399)
First week after summer school holidays		4.870^***^
		(10.18)
Second week after summer school holidays		5.125^***^
		(10.26)
Observations	4010	4010
Number of districts	401	401
*R*-squared	0.215	0.237

Linear fixed effects estimation. *t*-Statistics based on standard errors clustered on districts in parentheses. ^*^^*^^*^, ^*^^*^ and ^*^ refer to statistical significance at 1, 5 and 10% levels, respectively.

Model 1, which pools all holiday weeks together, masks that the effect is likely to vary and to increase over the holiday period. Model 2 is more appropriately specified as it allows the effect of the holiday season to vary week-by-week. We find that the effect increases in later weeks of the school holidays, consistent with our second hypothesis. The effect is close to zero in the first 2 weeks, rises from week three onwards, becomes statistically significant from week 4 onwards and increases to 4.15 cases per 100 000 people in week 6. The coefficients of the first and the second week after school holidays finish show that the increases in incidence rates brought about by the school holidays do not disappear but continue to rise to 5.13 cases per 100 000 people in the second week after school holidays end. In terms of substantive importance of this finding, an increase in the incidence rate of 4.15 cases in the final week of the holiday season equates to 44.7% of the average incidence rate across German districts during their sixth week of holidays, which is higher than the average incidence rate during the entire sample period and therefore represents the more appropriate benchmark against which the substantive effect size should be assessed so as not to overstate it. For the first and second week after holidays, the equivalent computation would suggest effects that equate to 46.0 and 45.3% of the average weekly incidence rate in those weeks.

In model 3, reported in Table 2, we allow the structural breaks to vary state-by-state but revert back to the simple Chow-type structural break model with only two dummy variables per state as otherwise we would have to report or visualize well over a hundred coefficients. We exclude the two states of Hamburg and Berlin since both are counted as consisting of only one district in our data, which would result in unreliable estimates in a district fixed effects specification. Table 2, in which we sort states by the point estimate of the holiday period dummy variable, shows large variation in the holiday effect on incidence rates across districts in different German states. Overall, we find that richer states are more likely to show relatively large effects, and we find that the increase in incidence rates associated with the holiday season tends to be larger in the Western German states than in the Eastern German states (Saxony, Thuringia, Saxony-Anhalt, Mecklenburg-Vorpommern and Brandenburg). Looking state by state, we find a statistically significant positive effect of the holiday period or the 2-week period after the end of holidays or of both in 12 of the 14 states included in model 3.

**Table 2:** School holiday effects, varying by state

*Model 3*	*During school holidays*	*After school holidays*
Incidence rate (*t* − 1)	0.373^***^	
	(14.31)	
Baden-Württemberg	3.903^***^	5.886^***^
	(6.027)	(6.561)
Bavaria	1.990^***^	4.500^***^
	(6.675)	(9.229)
Bremen	0.437^***^	0.919^**^
	(2.669)	(2.083)
Lower Saxony	0.304^***^	0.290^***^
	(7.873)	(6.932)
Hesse	0.224^***^	1.222^***^
	(3.097)	(5.520)
Schleswig-Holstein	0.0809^***^	0.194^***^
	(4.905)	(5.116)
Saxony	0.0739^***^	0.248^***^
	(3.755)	(3.847)
Thuringia	0.0454^**^	0.0665^**^
	(2.086)	(2.026)
Saxony-Anhalt	0.0435^**^	0.0280
	(2.142)	(1.559)
Rhineland-Palatinate	0.0640	0.448^***^
	(1.601)	(6.630)
Saarland	0.0635	0.217^**^
	(0.956)	(2.164)
Mecklenburg-Vorpommern	0.0231	0.187^**^
	(0.422)	(2.044)
North Rhine-Westphalia	−0.165	0.323
	(−0.579)	(1.205)
Brandenburg	−0.171^*^	0.137
	(−1.910)	(0.952)
Observations	4010	
Number of districts	401	
*R*-squared	0.236	

Linear fixed effects estimation. *t*-Statistics based on standard errors clustered on districts in parentheses. ^*^^*^^*^, ^*^^*^ and ^*^ refer to statistical significance at 1, 5 and 10% levels, respectively.

Overall, only two states in our sample do not show a statistically significant positive holiday effect: Brandenburg and North Rhine-Westphalia. Of these two cases, North Rhine-Westphalia appears to be an outlier. The state had high incidence rates before the holidays begun due to super-spreader events in a slaughterhouse of the Tönnies company in the districts of Gütersloh and Warendorf. If we drop the two districts of Gütersloh and Warendorf from the estimations then both coefficients of the holiday and post-holiday periods become statistically significantly positive for this state.

**Fig. 1:**
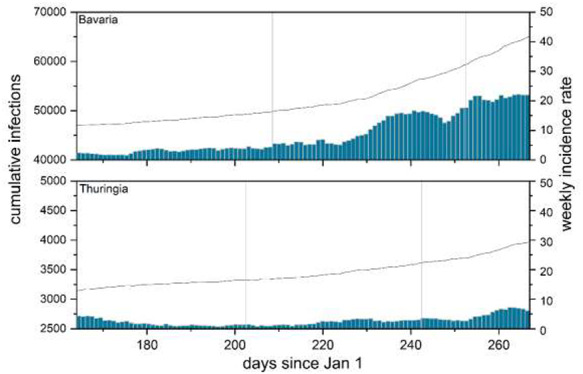
School holidays and infections in Bavaria and Thuringia. Note: Solid line indicates cumulative infections (left axis), bars weekly incidence rates (right axis). Vertical lines indicate summer school holiday period.

Figure 1 shows cumulative infection numbers (indicated by a solid line with their scale on the left-hand axis) and the weekly incidence rates (indicated by bars with their scale on the right-hand axis) for Bavaria, the richest German state bar the two city states of Bremen and Hamburg, and Thuringia, the state with one of the lowest average per capita income for each day between day 167 (10 June) and day 267 (23 September) of 2020. The vertical boundaries indicate the first and the last day of school holidays in these two states. As Model 3 has shown, the holiday season was associated with large increases in incidence rates relative to the trend in Bavaria, with much smaller increases relative to the trend in Thuringia. Figure 1 supports and illustrate these findings from our regression analysis.

Thuringia and Bavaria differ in many respects. Bayern is richer, more industrial, more urbanized, and it also hosts a larger share of foreign residents. In Table 3, we allow the effect of summer school holidays and the 2-week period after holidays to be conditioned by two variables, namely by average taxable income in a district as well as by the share of foreigners amongst a district’s residents. These variables are time-invariant for our sample, therefore we cannot estimate coefficients for these variables themselves in a model with district fixed effects. However, we can estimate the conditioning effect of these variables on the time-varying holiday variables.

**Table 3:** The conditioning effect of average taxable income and of the share of foreign residents

	*Model 4*
Incidence rate (*t* − 1)	0.365^*^*^^*^^
	(13.97)
Summer school holidays dummy	−4.064^*^*^^*^^
	(−3.696)
Post-holidays dummy	−5.734^*^^*^^*^
	(−4.174)
Holidays dummy^*^tax. income p.c.	0.273^*^^*^^*^
	(3.679)
Post-holidays dummy^*^tax. income p.c.	0.349^*^^*^^*^
	(3.665)
Holidays dummy^*^share foreign residents	0.0808^*^^*^ (2.261)
Post-holidays dummy^*^share foreign residents	0.397^*^^*^^*^ (5.785)
Observations	4010
Number of districts	401
*R*-squared	0.230

Linear fixed effects estimation. *t*-Statistics based on standard errors clustered on districts in parentheses. ^*^^*^^*^, ^*^^*^ and ^*^ refer to statistical significance at 1, 5 and 10% levels, respectively.

Model 4, reported in Table 3, shows a positive and statistically insignificant interaction effect between, respectively, average taxable income and the share of foreigners amongst a district’s residents with the dummy variables for school holidays and the post-holiday period, consistent with our fourth hypothesis. In substantive terms, the results from model 4 imply that the effect of the holiday period is almost six times stronger in districts with close to the highest share of foreign residents (increase in incidence rate of 6.72 cases per 100 000 people as opposed to an increase by 1.2 cases), while the effect of the 2-week post-holiday period is almost seven times stronger (increase in incidence rate of 20.5 cases per 100 000 people as opposed to an increase by 3.2 cases). The effect in the richest districts is eight times stronger than average during the holiday period and almost four times stronger than average in the post-holiday period.

## Discussion

### Main finding of this study

We have found that by the end of the holiday period the estimated effect equates to around 45% of the average incidence rate across German districts during their respective final week of holidays and their respective first 2 weeks after holidays end.

### What is already known on this topic

The RKI reports that the maximum of new infections for which a country abroad is stated as the most likely place of infection during Germany’s holiday season is around 49% in week 35 in mid-August with close to 45% in the 2 weeks either side of this maximum.

### What this study adds

Based on a research design that captures the effect of holidaying both within and outside Germany, our central estimates are slightly lower than the maximum of new infections for which a country abroad is stated as the most likely place of infection in reports to the RKI. Despite very different research designs, the two approaches find similar substantive average effects. Disaggregating the effect week-by-week, we find that the effect increases over the holiday period and does not revert back to what it was from before the holiday period in the 2 weeks after holidays end. We have demonstrated effects differ across German states with statistically significant holiday effects in at least 12 of the 14 German states with more than one district in our dataset. The stronger effects take place in the Western German states. Two main hypothesized reasons for this heterogeneity across German states were that the states with a stronger effect consist of districts that tend to be both richer and have a larger share of foreign residents amongst their population, both of which spurs holiday-related travel. Corroborating this, we have shown that the higher is per capita income and the higher the share of foreigners in a district, the larger the increases in the growth rate of infections.

### Limitations of this study

First, there are the well-known limitations of any ecological study like ours. Ideally, one would employ individual- rather than district-level data, however no such data exist and—due to privacy protection policies—cannot be collected. Second, we can only capture the effect of holiday-related travels triggered by public summer school holidays. Families with children of school-age in particular are dependent on school holidays for their holiday travel and the same holds for the employees of firms that close down for company holidays over that period. Thus, the majority of holiday travels will take place during school holidays. Yet, not all of holiday-related travel takes place during school holidays, which potentially biases downwards our estimate of the effect of holiday-related travels on Sars-CoV-2 infection.

## Conclusion

The impact that summer school holidays have had on incidence rates were entirely predictable and yet Germany’s public health authorities were not prepared for re-starting travel in the era of Covid-19.^16^ What they should have done was to significantly drive up testing facilities to compensate for the increase in infections and the reduced contact tracing capabilities. Eventually, Germany did introduce testing of returnees from particular high-risk destinations, but this came too late to prevent the significant increase in infections and, ironically, can further spread the virus if falsely negative tested individuals are lured into careless behavior.^17^ Governments should also improve digital tracing capabilities both within their territories but more importantly across borders if they wish to avoid travel restrictions. Germany in principle has a good tracing system being built on local infrastructure but the best tracing system cannot operate if infected individuals cannot recall with whom they had close contact during their holidays.^18^ Immunity passports to travel may also have to be reconsidered once vaccination becomes widely available despite their controversial nature.^19^

## Supplementary Material

Article_for_Journal_of_Public_Health_supplementary_document_fdab080

## Notes

**Thomas Plümper**, Professor of Quantitative Social Research

**Eric Neumayer**, Professor of Environment and Development
