# Supplementary material for: Fueling the Covid-19 pandemic: summer school holidays and incidence rates in German districts
Source: J Public Health (Oxf). 2021 Mar 26;43(3):e415–22. doi: 10.1093/pubmed/fdab080 (PMC8083696; doi:10.1093/pubmed/fdab080)
Supplement: Article_for_Journal_of_Public_Health_supplementary_document_fdab080 [file article_for_journal_of_public_health_supplementary_document_fdab080.docx]

*Supplementary document: Overview of the 2020 summer school holiday periods in the German states*

| state | holiday start | holiday end |
| --- | --- | --- |
| Baden-Württemberg | 30/07 | 12/09 |
| Bavaria | 27/07 | 07/09 |
| Berlin | 25/06 | 07/08 |
| Brandenburg | 25/06 | 08/08 |
| Bremen | 16/07 | 26/08 |
| Hamburg | 25/06 | 05/08 |
| Hesse | 06/07 | 14/08 |
| Mecklenburg-Vorpommern | 22/06 | 01/08 |
| Lower Saxony | 16/07 | 26/08 |
| North Rhine-Westphalia | 29/06 | 11/08 |
| Rhineland-Palatinate | 06/07 | 14/08 |
| Saarland | 06/07 | 14/08 |
| Saxony | 20/07 | 28/08 |
| Saxony-Anhalt | 16/07 | 26/08 |
| Schleswig-Holstein | 29/06 | 08/08 |
| Thuringia | 20/07 | 29/08 |
